# Cerebral Fat Embolism: Neuroprotective Goals in an Unusual Cause of Altered Mental Status

**DOI:** 10.7759/cureus.3054

**Published:** 2018-07-26

**Authors:** Keerthana K Kumar, Premkumar Nattanamai

**Affiliations:** 1 Neurology, University of Missouri, Columbia, USA

**Keywords:** cerebral, fat embolism, brain, star field, intracranial pressure, cerebral tissue oxygenation, cerebral fat embolism

## Abstract

Cerebral fat embolism syndrome is a rare, but potentially lethal, complication that may arise from long bone fractures and/or orthopaedic surgery. Neurological symptoms are variable, and clinical diagnosis is difficult. We report the case of a 75-year-old woman who developed cerebral fat embolism four days after a right hip arthroplasty. Maintenance of intracranial pressure monitoring (ICP) within normal limits and cerebral tissue oxygenation monitoring (PbtO2) over 20 mmHg prevented secondary brain injury and resulted in a gradual improvement of the patient’s sensorium. This case demonstrates that the use of ICP and PbtO2 monitoring defines optimal neuroprotective goals.

## Introduction

Fat embolism syndrome (FES) is a potentially catastrophic complication of long bone fractures. The incidence of fat embolism is highly variable and it is often a diagnosis of exclusion. The classic triad of symptoms that indicate FES are petechial skin rashes, hypoxemia, and neurological abnormalities, which usually occur within 24 - 72 hours after a long bone fracture. Cerebral fat embolism may occur in a subset of patients experiencing fat embolism. Cerebral fat embolism can manifest as focal deficits, seizures, altered mentation or coma [1].

Various mechanisms have been proposed to explain the pathophysiology of a cerebral fat embolism. Neuronal ischemia followed by cytotoxic edema occur in most patients with cerebral fat embolism. The ischemic changes typically occur in watershed areas, seen as a “star field” [2]. We report a patient who developed cerebral fat embolism after right hip arthroplasty.

## Case presentation

A previously healthy 75-year-old woman presented to her local hospital with a fractured right hip after suffering a fall at home. She was neurologically intact before the fall and denied any history of syncope or lower limb weakness. The following day (Day 2), a right hip hemiarthroplasty was performed. The procedure was uneventful. Soon after the surgery, the patient experienced deterioration of her respiratory status which required biphasic positive airway pressure (BiPAP). Chest X-ray was unremarkable. The patient was noted to have a urinary tract infection on admission and given ceftriaxone. Doxycycline was added empirically for possible community-acquired pneumonia. The following morning (Day 3), the patient was noted to have decreased responsiveness for which she was transferred to the intensive care unit (ICU). Dilaudid was discontinued in light of this worsening drowsiness. Magnetic resonance imaging (MRI) of the brain revealed subacute ischemia in the left basal ganglia and chronic small vessel ischemic disease (Day 3).

An electroencephalogram (EEG) on Day 4 demonstrated focal slowing with sharp bitemporal spikes. Levetiracetam (Keppra, UCB, Brussels, Belgium) was initiated for seizure prophylaxis. The following morning (Day 5), she had worsened to become unresponsive to painful stimuli. The patient was then intubated and transferred to our hospital for further management. Continuous electroencephalogram (EEG) monitoring was initiated. Antibiotic coverage escalated to include acyclovir, ampicillin, and vancomycin. Intravenous (IV) ceftriaxone and IV Keppra were continued. Lumbar puncture revealed normal opening pressure and clear cerebrospinal fluid (CSF). CSF protein and glucose were within normal limits, at 22 mg/dL and 91 mg/dL, respectively. There were no cells seen on CSF microscopy. An increased creatine kinase of 493 U/L and increased thyroid-stimulating hormone (TSH) level of 6.150 mcunit/mL were found on Day 5. She was started on 25 mcg levothyroxine. IV thiamine 500 mg was initiated for chronic alcoholism.

**Figure 1:**
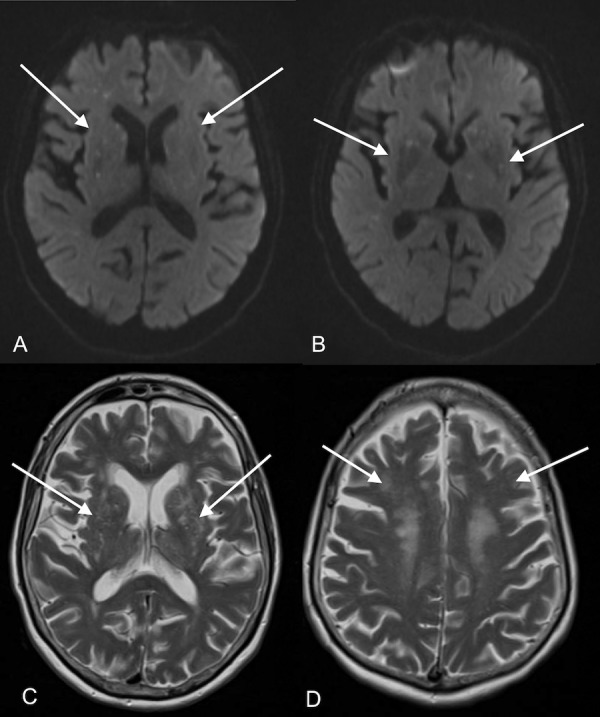
Magnetic resonance imaging (MRI) Diffusion-weighted (DW) MRI. (A, B) demonstrates multiple punctate foci of diffusion restriction in the gray-white junction; T2-weighted axial MRI (C, D) shows bilateral hyperintense areas in the periventricular white matter. Obtained six days after right hip arthroplasty.

Continuous EEG monitoring did not reveal any seizure activity. Antibiotics were reviewed after the CSF analysis to discontinue vancomycin and acyclovir (Day 6). Her pupils were 3 mm in diameter in the right eye and 2 mm in diameter in the left eye, which prompted a repeat computed tomography (CT) of the head, which did not reveal any new findings (Day 6). Brain MRI was repeated on Day 7 and revealed multiple punctate foci of diffusion restriction in the gray-white junction (Figure 1). Magnetic resonance angiography (MRA) was significant for chronic occlusion of the right internal carotid artery with distal middle cerebral artery reconstitution. Cerebral fat embolism (CFE) syndrome was suspected. Brain oxygen partial pressure (PbtO2) monitoring via Licox (Integra LifeSciences, Plainsboro, NJ, US) was initiated to optimize cerebral perfusion (Day 8). Subdural bolts were placed for continuous intracranial pressure monitoring via Camino (Integra LifeSciences, Plainsboro, NJ, US). Transthoracic echocardiogram (TTE) revealed no thrombus or a patent foramen ovale (Day 9). Patient’s sensorium improved with the eye opening in response to painful stimulus and was extubated (Day 9). She required bronchodilator therapy after extubation. Modified barium swallow demonstrated impaired pharyngeal clearing (the patient required 3-5 swallows to clear each bolus) and she was kept nil per os (NPO) (Day 12). She required 2L/min oxygen via nasal cannula to maintain saturation. Intracranial pressure monitoring and PbtO2 monitoring were discontinued once cerebral oxygenation and intracranial pressure were within physiological limits. She remained drowsy post-extubation. She opened her eyes to verbal stimuli but did not speak coherently. Her sensorium improved gradually and passed modified barium swallow (Day 15). She was discharged the following day (Day 16).

## Discussion

The incidence of fat embolism in patients with long bone fractures depends on the location and the severity of fracture [3-4]. Due to the absence of a gold standard to diagnose fat embolisms, the true incidence of fat embolism may be underrepresented.

Our patient developed cerebral fat embolism syndrome after a long bone fracture and total hip arthroplasty. The incidence of fat embolisms post-orthopedic surgery has been investigated, and the most prominent manifestation is acute hypoxia, present in 96% of cases [3]. The incidence of fat embolism is higher after long bone fractures than after hip arthroplasty [4-5]. A clinical diagnosis of cerebral fat embolism can be substantiated with diffusion-weighted magnetic resonance imaging (DW-MRI) and axial T2 findings. It has been postulated that changes seen on DWI reflect early cytotoxic edema after fat embolism, and T2 hyperintensities signify vasogenic edema, known to occur later in the course of fat embolism syndrome [2].

Cerebral fat emboli can often cause the brain to appear edematous and shows an inflammatory reaction while the numerous petechiae can cover the surface of the brain. It has been hypothesized that the volume of diffusion restriction on an initial MRI may be able to predict outcomes [6-8]. A fulminant fat embolism is characterized by the occlusion of the microvasculature by fat emboli, resulting in microinfarctions and hemorrhage [9].

Stroke was also considered as a differential diagnosis since the right internal carotid artery showed features of chronic occlusion. However, considering the inciting event and bilateral nature of the MRI findings in our patient, a fat embolism seemed more likely than an embolic stroke. Our patient experienced significant neurological deterioration due to the fat embolism and recovered to baseline neurological status 15 days after arthroplasty for long bone fracture.

Neurological deficits after a fat embolism occur when intracranial hypertension persists. The progressive increase in intracranial pressure can cause herniation and brain death [10]. Neuromonitoring helped in the prognostication of these patients and also enabled optimum neuro-protective care. Brain tissue oxygenation (PbtO2) was maintained over 20 mmHg. Maintenance of intracranial pressure (ICP) within physiological limits also prevented secondary brain injury. Since increased ICP can trigger a vicious cycle of ischemia and edema, we recommend monitoring of ICP and cerebral oxygenation for precisely defining neuroprotective goals [11-12]. Our patient's recovery was facilitated by adequate control of intracranial pressure guided by intracranial pressure monitoring and optimization of cerebral oxygenation based on brain tissue oxygenation monitoring.

## Conclusions

Fat embolism syndrome is a rare cause of altered mental status. The presence of fracture and/or orthopedic surgery in combination with clinical and brain MRI findings help in the diagnosis of cerebral fat embolism syndrome. The use of intracranial pressure monitoring and cerebral tissue oxygenation monitoring clearly define neuro-protective targets for optimum perfusion and intracranial pressure control.
